# Mechanism of Action of Therapeutic Exercise in Rotator Cuff Tendinopathy: What Does Elastography Add?

**DOI:** 10.3390/jcm15031015

**Published:** 2026-01-27

**Authors:** Irene Pérez-Porta, Claudia de la Fuente-Escudero, Ángel Luis Bueno-Horcajadas, Elia Pérez-Fernández, Fernando García-Pérez, María Velasco-Arribas, Mariano Tomás Flórez-García

**Affiliations:** 1 Department of Physical Medicine and Rehabilitation, Hospital Universitario Fundación Alcorcón, 28922 Madrid, Spain; ipporta@salud.madrid.org (I.P.-P.); marianotomas.florez@salud.madrid.org (M.T.F.-G.); 2 International Doctoral School, Universidad Rey Juan Carlos, 28933 Mostoles, Madrid, Spain; 3 Department of Radiology, Hospital Universitario Fundación Alcorcón, 28922 Madrid, Spain; angelluis.bueno@salud.madrid.org; 4 Statistics and Data Analysis Unit, Hospital Universitario Fundación Alcorcón, 28922 Madrid, Spain; eliapf@salud.madrid.org; 5 Research Unit, Hospital Universitario Fundación Alcorcón, 28922 Madrid, Spain

**Keywords:** sonoelastography, shear wave elastography, rotator cuff tendinopathy, supraspinatus muscle, exercise, stiffness

## Abstract

**Background/Objectives**: In rotator cuff-related shoulder pain (RCRSP) without associated tendon rupture, therapeutic exercise is one of the main treatment strategies; however, the mechanisms through which it exerts its effects remain poorly understood. The aim of this study was to analyze the role of two potential mechanisms of action: increases in muscle strength and changes in the microarchitecture of the supraspinatus muscle. **Methods**: This prospective study included 39 patients with RCRSP treated with a strengthening exercise program. Muscle strength was assessed using dynamometry, and supraspinatus muscle stiffness was evaluated using shear wave elastography (SWE) at baseline and after 6 months of exercise. These measurements were correlated with clinical and perceived improvement in the patients. **Results**: Thirty-seven patients completed follow-up. At 6 months, 67.6% of participants reported being much better or fully recovered, 29.7% reported being somewhat better, and only one patient (2.7%) reported worsening. Overall, the increase in muscle strength was small. In participants reporting marked improvement or full recovery, strength gains were slightly greater, but a significant increase in supraspinatus muscle stiffness was observed. In contrast, participants with mild improvement or worsening showed smaller strength gains and no changes in muscle stiffness. **Conclusions:** Strength gains following strengthening exercises in RCRSP are small and of limited clinical relevance. In contrast, increased supraspinatus muscle stiffness assessed by SWE was observed after the exercise intervention and may reflect exercise-related adaptations; however, its biological meaning should be interpreted with caution.

## 1. Introduction

Shoulder pain is a common symptom [1] and can significantly impair quality of life [2]. In most cases, symptoms are associated with involvement of the rotator cuff muscles, particularly the supraspinatus muscle [3,4]. In rotator cuff-related shoulder pain (RCRSP) without associated tendon rupture, the most recent clinical practice guidelines recommend exercise programs as the primary treatment [5,6]. Although numerous studies have been conducted, there is still limited information regarding which types of exercise are most effective and, more importantly, about the mechanisms through which exercise leads to clinical improvement [7]. In a recent systematic review [8], 110 clinical trials investigating exercise interventions in RCRSP were analyzed, identifying up to 32 different proposed mechanisms of action.

In the case of strengthening programs—the most commonly used intervention in RCRSP—many authors have traditionally considered increases in muscle strength to be the most relevant mechanism. However, this remains a matter of debate, as previous studies have shown that the relationship between strength gains and clinical improvement is often weak and inconsistent [9,10]. In addition to strength gains, it has also been suggested that exercise performed repeatedly over several months may improve the mechanical properties and microarchitecture of the supraspinatus tendon or muscle tissue [11]. Changes in the properties of muscle tissue and tendon are difficult to assess, detect, and quantify. Several studies [12,13,14] have attempted to analyze treatment effects on the microarchitecture of the supraspinatus tendon and muscle tissue using advanced imaging techniques such as magnetic resonance imaging (MRI) or elastography. Elastography allows quantification of biological tissue stiffness with adequate intra- and inter-observer reliability [15]. In particular, shear wave elastography (SWE) does not depend on the pressure applied by the operator with the ultrasound transducer, as it is based on acoustic micro-impulses generated by the ultrasound system itself. This technique measures, in meters per second (m/s), the velocity at which a transverse shear wave propagates through the supraspinatus tendon or muscle, a parameter that correlates with tissue elasticity. SWE has been suggested to be particularly useful for detecting and monitoring subtle changes in tendon and muscle microstructure in response to treatment [11].

At the time this study was designed (2022), two prospective studies had been published analyzing the potential effects of strengthening exercises on supraspinatus tendon microarchitecture using advanced imaging techniques. In both studies, imaging was performed at baseline and after completion of the exercise program. One study used MRI [12], and the other employed SWE [13]; however, neither demonstrated significant post-treatment changes. In contrast, another study [14], in which SWE was applied within the supraspinatus muscle, reported changes in stiffness values before and after treatment that correlated with clinical improvement. These findings suggested that elastographic assessment focused on the supraspinatus muscle, rather than the tendon, might be more sensitive for detecting exercise-induced changes.

The aim of this study was to analyze the potential role of two exercise-related mechanisms proposed in RCRSP: increases in muscle strength and changes in supraspinatus microarchitecture. To this end, muscle strength was assessed using dynamometry, and supraspinatus muscle stiffness was evaluated using SWE before and after a 6-month strengthening exercise program. The observed changes in both variables were then correlated with the degree of patient-perceived clinical improvement, shoulder pain and function.

## 2. Materials and Methods

### 2.1. Study Design

A prospective observational study was conducted in a subgroup of patients participating in a randomized controlled trial (RCT). The primary objective of the main RCT was to evaluate whether supplementing face-to-face physiotherapist instruction with a digital application including multimedia animations improved clinical outcomes.

The original trial included 154 patients (80 in the control group and 74 in the experimental group) and was conducted in accordance with the Consolidated Standards of Reporting Trials (CONSORT) guidelines. The methodology of the main trial has been described in detail previously [16,17].

To explore potential mechanisms underlying the effects of exercise, a subgroup of 39 patients from the RCT who had not previously undergone shoulder ultrasound assessment was selected. These participants underwent conventional shoulder ultrasound to exclude full-thickness tendon tears. Supraspinatus muscle stiffness was assessed using SWE, and shoulder abduction strength was measured using dynamometry. All measurements were performed at baseline (prior to initiation of the exercise program) and at 6 months (end of follow-up).

The study was approved by the Ethics Committee of Hospital Universitario Fundación Alcorcón, Madrid, Spain (approval code CI18/16). This study represents a secondary analysis of a completed randomized controlled trial registered at ClinicalTrials.gov (NCT05770908; first posted on 14 March 2023).

### 2.2. Patient Recruitment

This single-center study was conducted at a tertiary care hospital between 23 April 2023 and 1 December 2024. Patients were recruited by three rehabilitation physicians.

Inclusion criteria were: age between 18 and 80 years; unilateral shoulder pain located in the anterior and/or lateral region for at least 3 months; pain triggered or exacerbated by shoulder elevation, internal rotation, and/or ipsilateral side-lying position during sleep; at least one positive subacromial impingement test (Neer, Hawkins–Kennedy, or empty can/Jobe test); pain intensity ≥3/10 on the numerical pain rating scale (NPRS) at rest, during activity, and/or at night; access to an internet-connected device (mobile phone, tablet, or computer); and ability to provide written informed consent in Spanish.

Exclusion criteria included: history of major trauma as the origin of pain; previous surgery of the neck, shoulder, or elbow within the preceding 6 months; alternative causes of shoulder pain (e.g., instability, calcific tendinitis, osteoarthritis, polymyalgia rheumatica, osteonecrosis, neuralgic amyotrophy, septic arthritis); signs or symptoms of cervical pain or radiculopathy; limitation of passive shoulder range of motion suggestive of adhesive capsulitis; imaging evidence of full-thickness tendon tear; cognitive impairment affecting cooperation; severe psychiatric disorders; severe neurological diseases (e.g., stroke, multiple sclerosis, Parkinson’s disease); or other relevant medical conditions affecting the ipsilateral upper limb.

Baseline assessments were recorded after recruitment.

### 2.3. Intervention

All participants completed a therapeutic exercise program supervised by two physiotherapists with more than five years of clinical experience. The protocol consisted of five face-to-face sessions on alternate days over three weeks, with two additional follow-up sessions at weeks 6 and 12 for program review and progression.

The program included progressive strengthening exercises targeting the rotator cuff (abductors and external rotators) and periscapular musculature (protractors and retractors). Posterior capsule stretching exercises were added in selected cases. Exercise progression was guided by pain intensity (not exceeding 4/10) and, in the absence of pain, by perceived exertion (≥6/10).

All participants received printed exercise instructions. Patients in the experimental group additionally had access to a web-based platform with instructional videos. The intervention was reported in accordance with the Consensus on Exercise Reporting Template (CERT). Full details of the exercise program are provided in the Supplementary Materials, and an extended description has been published previously.

### 2.4. Adherence

Adherence to the exercise program was monitored using a patient exercise diary, in which participants prospectively recorded completed exercise sessions throughout the intervention period. Detailed adherence data are provided in the Supplementary Materials.

### 2.5. Ultrasound Assessment

Shoulder ultrasound examinations were performed by a musculoskeletal radiologist with more than 25 years of experience using a Canon Aplio i600 ultrasound system (Canon Medical Systems, Tustin, CA, USA) equipped with a 14 MHz linear transducer. A standardized protocol was followed to systematically evaluate the supraspinatus, infraspinatus, teres minor, subscapularis tendons, and the long head of the biceps tendon in longitudinal and transverse planes. Tendon integrity, echogenicity, thickness, and the presence of bursal fluid were assessed.

Detailed ultrasound methodology, including patient positioning, probe orientation, machine settings, artifacts, and representative images, is provided in the Supplementary Materials.

### 2.6. Shear Wave Elastography

Supraspinatus muscle stiffness was assessed using SWE with the same ultrasound system and transducer. The region of interest (ROI) was placed in the thickest portion of the muscle, aligned with the longitudinal fiber orientation. Three consecutive measurements were obtained at rest and during maximal isometric contraction.

**Figure 1:**
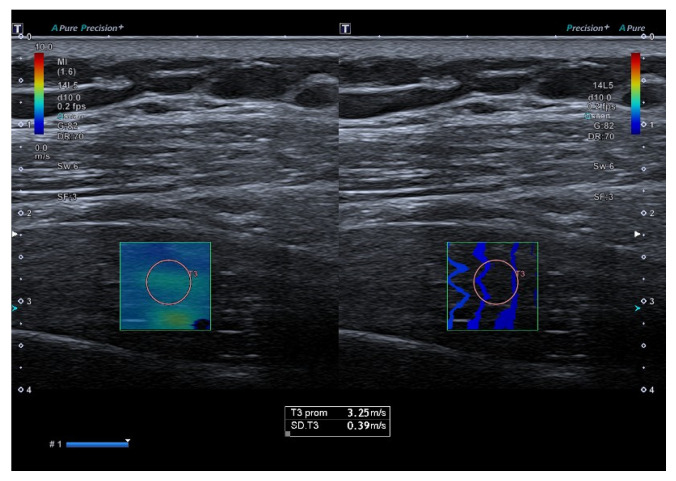
Shear wave elastography of the supraspinatus muscle at rest.

For resting SWE (SWE-R) and force-related measurements, the final value was calculated as the weighted mean of the three measurements, whereas for contraction SWE (SWE-C), the maximum value was retained (Figure 1). A detailed description of the SWE procedure, including physical principles, device parameters, artifact control, positioning criteria, and representative images, is available in the Supplementary Materials.

All SWE measurements were performed by the same evaluator, who was not blinded to the assessment time point.

### 2.7. Isometric Strength Measurement

**Figure 2:**
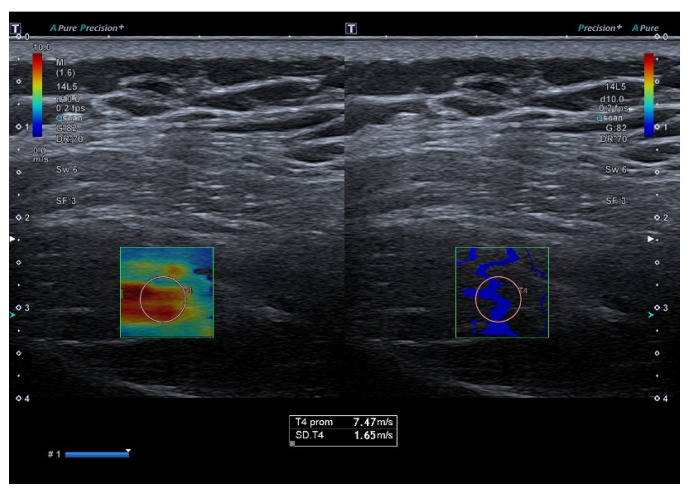
Shear wave elastography of the supraspinatus muscle at isometric muscle contraction.

Isometric glenohumeral abduction strength was assessed using a digital dynamometer (Carp Spirit, Bourgogne, France), following the same positioning protocol used for SWE measurements. Three trials were performed, and the mean value was used for statistical analysis (Figure 2). Additional methodological details are provided in the Supplementary Materials.

No additional reliability testing was performed for shear wave elastography (SWE) or isometric shoulder strength measurements in the present study, as the reliability of these methods has been well established in previous research. Multiple studies have demonstrated good to excellent intra- and inter-observer reliability for SWE when assessing supraspinatus muscle stiffness [18,19].

Similarly, maximal isometric upper limb strength assessed using handheld dynamometry has been shown to be valid and highly reliable, with excellent intra- and inter-rater reliability for major shoulder movements, including abduction [20].

### 2.8. Clinical and Perceived Improvement

Clinical improvement was evaluated through changes in pain and functional limitation. Functional status was assessed using the Spanish version of the Shoulder Pain and Disability Index (SPADI; 0–100%, with higher scores indicating greater disability). Pain intensity over the previous week was assessed at rest, during movement, and at night using the NPRS; 0–10.

Clinically relevant improvement thresholds were based on data from the previously published RCT, which reported mean reductions at 24 weeks of approximately 25 points in SPADI, 2 points in resting NPRS, and 3 points in movement and nighttime NPRS.

Based on these thresholds, patients were classified as “clinically improved” or “not improved” for the purpose of subgroup analyses.

Global perceived improvement was assessed using the Patient Global Impression of Improvement (PGI-I) scale (1 = much worse; 7 = much better).

All assessments were conducted at the Hospital. Baseline and 24-week assessments were performed by rehabilitation physicians, while assessments at weeks 3, 6, and 12 were conducted by the physiotherapists responsible for the exercise program.

Demographic data collected included age, height, weight, body mass index (BMI), sex, dominance, affected side, and symptom duration. Measures to minimize selection and measurement bias included consistent application of inclusion criteria, use of validated instruments, and performance of all measurements by the same observer.

### 2.9. Statistical Analysis

All analyses were performed using STATA v17.0 (StataCorp LLC, College Station, TX, USA). Descriptive statistics are presented as means with standard errors (SE) or medians with interquartile ranges (IQR), as appropriate.

To assess the effect of the intervention on supraspinatus stiffness (SWE-R and SWE-C) and muscle strength, and to examine whether these effects differed according to clinical and perceived improvement, linear mixed-effects models were constructed for each outcome. Fixed effects included time (baseline vs. post-treatment), group (“improvement” vs. “no improvement”), and their interaction (time × group). A random intercept for each participant was included to account for within-subject correlation.

No imputation methods were applied, as no missing data were observed.

The interaction term was used to test whether changes from baseline differed between groups. Estimated marginal means and mean changes from baseline with 95% confidence intervals (CI) were derived from the models. Statistical significance was set at *p* < 0.05.

## 3. Results

### 3.1. Baseline Data

All 39 patients included in the study completed the exercise program. The mean age of the sample was 55.6 ± 10.2 years, and the BMI was 27.3 ± 4.4 kg/m^2^. Most patients were women (64.1%). Pain laterality was right-sided in 53.8%, and the dominant side coincided with the affected shoulder in 51.3% of participants. Educational level was evenly distributed: 25.6% had primary education, 35.9% secondary education, and 38.5% university studies. Regarding previous treatments, 51.3% had used analgesics, and only 5.1% had received physiotherapy (Table 1).

Baseline scores indicated moderate pain intensity: the NPRS at rest averaged 3.5 ± 2.5, the NPRS during movement 5.9 ± 2.0, and the nighttime NPRS 6.0 ± 2.8. The SPADI disability score showed a mean of 52.9 ± 20.5, reflecting a moderate level of shoulder functional limitation (Table 1).

SWE-R showed a mean value of 3.78 ± 0.70 m/s. Compared with previously published normative values, 46% of patients presented values > p75, 38% values within the normal range and 16% < p25. Intra-individual variability at rest (SWE-R SD) was 0.56 ± 0.21 m/s. SWE-C was markedly higher, with a mean value of 7.82 ± 1.02 m/s. A total of 49% of patients showed values > p75, 27% within the normal range and 24% < p25 [21]. Variability during contraction (SWE-C SD) was 2.15 ± 0.78 m/s, indicating substantial dispersion between individuals. Baseline muscle strength was 5.54 ± 3.62 kg, with high inter-individual variability (Table 2).

### 3.2. Supraspinatus Muscle Stiffness Using SWE and Muscle Strength Before and After a Therapeutic Exercise Program

**Figure 3:**
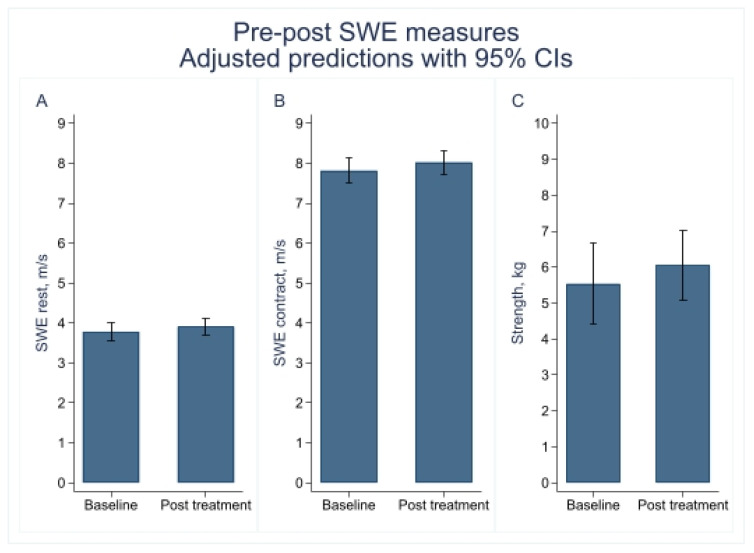
Pre–post changes in supraspinatus shear wave elastography measures and muscle strength. (**A**) Adjusted mean shear wave elastography (SWE) values at rest (m/s) with 95% confidence intervals. (**B**) Adjusted mean SWE values during maximal isometric contraction (m/s) with 95% confidence intervals. (**C**) Adjusted mean muscle strength values (kg) with 95% confidence intervals at baseline and post-treatment. Abbreviations: CI, confidence interval; m/s, meters per second; SWE, shear wave elastography.

Mixed-effects model analyses showed no statistically significant time effects for any of the variables assessed. SWE-R increased slightly from 3.78 to 3.91 m/s (*p* = 0.105), and SWE-C rose from 7.82 to 8.03 m/s (*p* = 0.285), but these changes were not significant. Muscle strength showed a modest improvement from 5.54 to 6.05 kg (*p* = 0.209) (Table 3 and Figure 3).

### 3.3. Supraspinatus Muscle Stiffness Using SWE and Muscle Strength Before and After a Therapeutic Exercise Program According to Clinical Improvement

At 24 weeks, clinically relevant improvement was observed in 17 participants for functional status, in 21 for resting pain, in 23 for pain during movement, and in 22 for nighttime pain.

**Figure 4:**
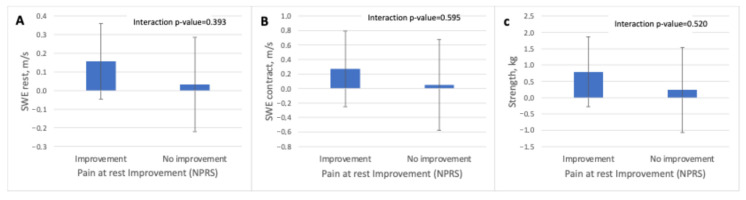
Adjusted pre–post changes in supraspinatus muscle elasticity and glenohumeral abduction strength according to perceived improvement in pain at rest. Adjusted differences from baseline (mean and 95% confidence intervals) for (**A**) shear wave elastography at rest, (**B**) shear wave elastography during maximal isometric contraction, and (**C**) muscle strength, stratified by perceived improvement in pain at rest assessed with the Numeric Pain Rating Scale (NPRS). Interaction *p*-values correspond to the time × group effect derived from linear mixed-effects models. Abbreviations: kg, kilograms; m/s, meters per second; NPRS, Numeric Pain Rating Scale; SWE, shear wave elastography.

For resting pain (NPRS rest), the mixed-effects models showed no significant time-by-group interaction for any of the variables assessed. Participants who reported improvement exhibited small increases in supraspinatus elasticity in both resting and contracted states (SWE-R: +0.16 m/s; SWE-C: +0.27 m/s), whereas the non-improvement group showed minimal changes (SWE-R: +0.03 m/s; SWE-C: +0.05 m/s). Similarly, muscle strength increased slightly in the improvement group (+0.79 kg) and showed only a small variation in the non-improvement group (+0.24 kg) (Table 4 and Figure 4).

**Figure 5:**
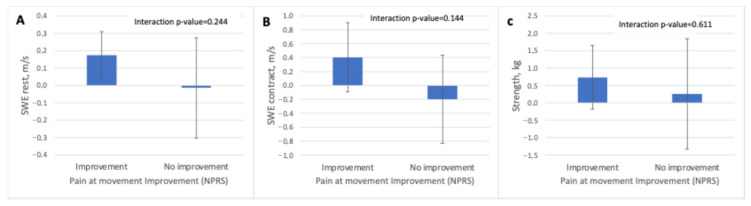
Treatment effects on supraspinatus shear wave elastography measures and muscle strength according to perceived improvement in pain during movement. Adjusted differences from baseline (mean and 95% confidence intervals) for (**A**) shear wave elastography at rest, (**B**) shear wave elastography during maximal isometric contraction, and (**C**) muscle strength, stratified by perceived improvement in pain during movement assessed with the Numeric Pain Rating Scale (NPRS). Interaction *p*-values correspond to the time × group effect derived from linear mixed-effects models. Abbreviations: kg, kilograms; m/s, meters per second; NPRS, Numeric Pain Rating Scale; SWE, shear wave elastography.

For movement pain (NPRS move), the mixed-effects models did not reveal a significant time-by-group interaction for any variables. Participants who reported improvement showed small increases in supraspinatus elasticity in both resting and contracted states (SWE-R: +0.17 m/s; SWE-C: +0.40 m/s), while the non-improvement group demonstrated minimal or slightly negative changes (SWE-R: −0.01 m/s; SWE-C: −0.20 m/s). Muscle strength also increased modestly in the improvement group (+0.74 kg), whereas changes were negligible among those without perceived improvement (+0.26 kg) (Table 4 and Figure 5)

**Figure 6:**
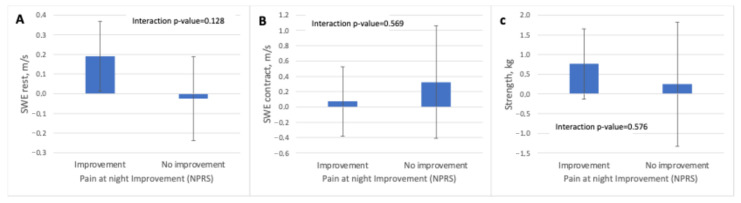
Treatment effects on supraspinatus shear wave elastography measures and muscle strength according to perceived improvement in nighttime pain. Adjusted differences from baseline (mean and 95% confidence intervals) for (**A**) shear wave elastography at rest, (**B**) shear wave elastography during maximal isometric contraction, and (**C**) muscle strength, stratified by perceived improvement in nighttime pain assessed with the Numeric Pain Rating Scale (NPRS). Interaction *p*-values correspond to the time × group effect derived from linear mixed-effects models. Abbreviations: kg, kilograms; m/s, meters per second; NPRS, Numeric Pain Rating Scale; SWE, shear wave elastography.

For nighttime pain (NPRS night), the analyses again showed no significant time-by-group interaction for any muscular outcome. In the improvement group, small increases were noted in SWE-R (+0.19 m/s) and minor positive changes in the contracted condition (+0.07 m/s). In contrast, the non-improvement group demonstrated minimal or slightly negative changes (SWE-R: −0.02 m/s; SWE-C: +0.32 m/s). Strength increased modestly in the improvement group (+0.76 kg) and only slightly in the non-improvement group (+0.25 kg). As with the other pain domains, no variable demonstrated a significant differential response between groups (Table 4 and Figure 6).

**Figure 7:**
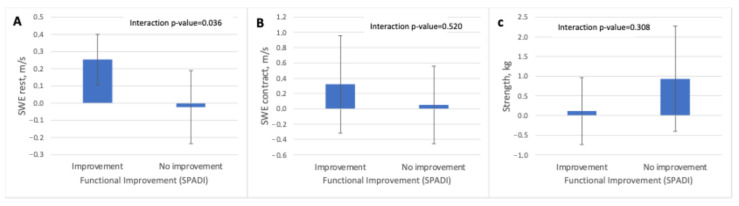
Treatment effects on supraspinatus shear wave elastography measures and muscle strength according to perceived functional improvement. Adjusted differences from baseline (mean and 95% confidence intervals) for (**A**) shear wave elastography at rest, (**B**) shear wave elastography during maximal isometric contraction, and (**C**) muscle strength, stratified by perceived functional improvement assessed with the Shoulder Pain and Disability Index (SPADI). Interaction *p*-values correspond to the time × group effect derived from linear mixed-effects models. Abbreviations: kg, kilograms; m/s, meters per second; SPADI, Shoulder Pain and Disability Index; SWE, shear wave elastography.

For shoulder disability (SPADI), mixed-effects models revealed a significant time-by-group interaction for resting stiffness (SWE-R; *p* = 0.036), while no significant interactions were observed for SWE-C. Participants who reported improvement showed an increase in resting supraspinatus stiffness (+0.25 m/s), whereas the no-improvement group experienced a negligible and non-significant change (−0.02 m/s). Changes in SWE-C were small and non-significant in both groups (improvement: +0.32 m/s; no improvement: +0.05 m/s). Muscle strength showed minimal change in the improvement group (+0.11 kg) and a larger but imprecise increase in the no-improvement group (+0.93 kg). (Table 4 and Figure 7).

### 3.4. Supraspinatus Muscle Stiffness Using SWE and Muscle Strength Before and After a Therapeutic Exercise Program According to Perceived Improvement

Two participants did not provide global perceived improvement data at the 24-week follow-up. At 24 weeks, 97.3% of the participants reported some degree of improvement after the exercise program: 11 patients (29.7%) felt “somewhat better,” 20 (54.1%) “much better,” and 5 (13.5%) reported being “fully recovered.” Only one patient (2.7%) reported worsening. Mixed-effects analyses examining changes according to perceived improvement showed no significant time-by-group interactions for any of the stiffness or strength measures. Changes in SWE measures and muscle strength did not differ between participants with greater versus lesser perceived improvement, suggesting that subjective recovery was not associated with measurable variations in supraspinatus stiffness or strength.

Participants who reported being much better or totally recovered displayed small, non-significant increases in resting stiffness (SWE-R: +0.16 m/s), contraction stiffness (SWE-C: +0.27 m/s), and strength (+0.71 kg)

Similarly, participants reporting lower levels of improvement (from much worse to somewhat better) showed minimal changes from baseline in all variables, with no significant effects detected.

**Figure 8:**
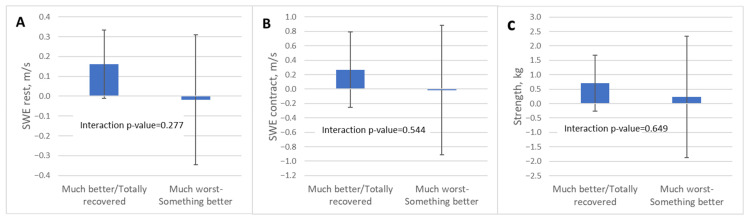
Relationship between perceived global improvement and supraspinatus shear wave elastography measures and muscle strength. Adjusted mean differences from baseline (mean and 95% confidence intervals) for (**A**) shear wave elastography at rest, (**B**) shear wave elastography during maximal isometric contraction, and (**C**) muscle strength, stratified by levels of perceived global improvement assessed with the Patient Global Impression of Improvement scale. Participants were grouped as “much better/totally recovered” versus “much worse/something better”. Interaction *p*-values correspond to the time × group effect derived from linear mixed-effects models. Abbreviations: CI, confidence interval; kg, kilograms; m/s, meters per second; SWE, shear wave elastography.

These results are presented in Table 5 and Figure 8.

Adherence to the exercise program was high overall, with adherence rates of 77.6%, 71.0%, 59.4%, and 69.0% at weeks 3, 6, 12, and 24, respectively, and a median global adherence of 68% (interquartile range [IQR]: 54–83%). No statistically significant differences in adherence were observed between groups according to either clinical improvement or perceived improvement. Detailed adherence data are provided in Table S2 and Supplementary Figures S2–S4.

## 4. Discussion

Exercise programs are among the most widely used and potentially effective treatments for individuals with RCRSP [7]. However, optimizing exercise prescription parameters requires a better understanding of the mechanisms through which exercise produces clinical improvement [22]. Most authors have proposed neuromuscular mechanisms, such as increases in muscle strength or improvements in motor control, to explain the clinical benefits perceived by many patients undergoing strengthening exercise programs. While it is reasonable to assume that strength gains play an important role in this context, studies have consistently shown that the relationship between muscle strength and clinical improvement is weak or inconsistent [11]. In a large study involving 1407 patients with knee and hip osteoarthritis treated with strengthening exercises [23], only changes in knee strength were statistically associated with improvements in pain and function; however, quadriceps strength gains accounted for only 2% of the observed clinical improvement. In RCRSP, clinical trials assessing strength as an intermediate outcome following exercise interventions have reported variable and sometimes inconsistent results [9]. Moreover, several studies have demonstrated that higher exercise intensity or greater strengthening exercise doses are not necessarily associated with better clinical outcomes [10].

In line with this evidence, recent conceptual frameworks have questioned the assumption that exercise-based rehabilitation primarily acts by correcting isolated physical deficits such as muscle weakness. In this context, Powell et al. [24] emphasized that although exercise remains effective for musculoskeletal pain, its benefits should be understood within a broader model of supported and personalized self-management rather than being framed solely as a strategy to restore strength. While strength remains relevant for general health and function, this perspective highlights the importance of also prioritizing reassurance, gradual reactivation, and avoidance of over-medicalization and over-diagnosis in rehabilitation programs.

Beyond strength-related adaptations, a second potential mechanism that has been proposed is whether prolonged exercise performed with an adequate dose and intensity may induce structural or mechanical adaptations in muscle or tendon tissue. Such adaptations may involve remodeling processes, including changes in collagen fiber organization or muscle tissue composition, which could influence muscle quality and function. Structural adaptations of tendon tissue have been described in isolated cases at the level of the supraspinatus tendon [25] and more frequently in other tendinopathies, such as the Achilles or patellar tendon, particularly following long-term exercise interventions [26]. However, structural tissue changes do not always parallel symptom evolution, as tendon normalization may occur without symptom resolution [27], and clinical recovery may also be observed in the absence of detectable structural changes [28].

Several studies have attempted to evaluate the effects of exercise on supraspinatus tendon and muscle microarchitecture using ultrasound and MRI. Østerås et al. [12] reported a case series of six patients who underwent MRI before and after a 3-month exercise program, observing no changes in supraspinatus tendon thickness despite significant clinical improvement. Similarly, Brage et al. [13] studied 23 patients using elastography at baseline and after 3 months of exercise and found no structural changes in the supraspinatus tendon. In contrast, another study [14] in which SWE was applied within the supraspinatus muscle reported changes in elastography parameters that correlated with improvements in pain and function following conservative treatment, although that intervention was not based exclusively on exercise. For this reason, elastographic assessment in the present study was focused on the supraspinatus muscle rather than the tendon, as it appeared to be more sensitive for detecting microarchitectural changes after several months of exercise. Subsequently, three additional studies have been published. Two focused on the tendon [11,29], suggesting that exercise programs may indeed induce detectable changes in supraspinatus tendon microarchitecture, while a third recent study [30] analyzed the effects of an exercise program on supraspinatus muscle microarchitecture and also reported positive changes.

Against this background, in the present study, a 6-month exercise program resulted in an increase in shear wave propagation velocity (m/s), indicating increased muscle stiffness. The interpretation of this finding is highly context-dependent, as increased supraspinatus muscle stiffness in individuals with RCRSP may have different implications depending on the degree of tendon and muscle involvement and on the temporal evolution of tissue changes.

In individuals with RCRSP associated with full-thickness supraspinatus tendon tears, secondary changes occur at the muscle level. Rosskopf et al. [19] described the temporal sequence of these changes. In early stages (Goutallier stages 0–III), fatty infiltration without fibrosis and muscle atrophy are observed. Fatty infiltration and muscle atrophy reduce shear wave propagation velocity, reflecting decreased muscle stiffness. Over time, however, fibrosis may develop, leading to increased stiffness (higher shear wave velocity), which represents progressive and irreversible muscle deterioration (Goutallier stage IV). Slowly developing increases in muscle stiffness in this context reflect fibrosis and irreversible tissue damage. Such muscle and tendon degeneration is also associated with an increased risk of re-tear following rotator cuff repair due to poor muscle quality [31].

By contrast, in RCRSP without major structural changes—i.e., with a normal tendon or mild tendinosis—the muscle is typically structurally preserved. In this scenario, an increase in muscle stiffness detected by elastography after several months of exercise is interpreted as an increase in muscle density [32], reflecting improved muscle quality. This phenomenon is analogous to differences observed in elastography values between asymptomatic men and women with normal or mildly tendinotic tendons, where men typically exhibit higher muscle density and therefore higher stiffness values [33].

The most relevant finding of this study is the correlation between increases in elastography-derived SWE and clinical improvement. In the subgroup of patients reporting marked improvement, the supraspinatus muscle exhibited increased muscle density, although statistical significance was not reached due to the small sample size. In contrast, participants with mild improvement or worsening showed no changes in shear wave velocity. Increased muscle stiffness measured by SWE, expressed as shear wave propagation velocity (m/s) through the supraspinatus muscle, may therefore represent an objective marker of improvement, indicating that exercise has induced positive changes in muscle quality.

Although the observed increase in supraspinatus muscle stiffness after the exercise program is interpreted as a favorable adaptation related to improved muscle quality and load tolerance in this clinical context, shear wave elastography cannot directly distinguish between adaptive and maladaptive tissue changes. Increased stiffness may also reflect unfavorable processes, such as fibrosis, in other pathological scenarios. However, this risk was minimized by excluding participants with full-thickness tendon tears or advanced fatty muscle degeneration, resulting in a relatively homogeneous sample with preserved muscle structure. Nevertheless, SWE findings should be interpreted cautiously and within the appropriate clinical context, underscoring the need for future studies combining elastography with complementary imaging techniques.

Exercise programs have demonstrated clinical effectiveness comparable to corticosteroid injections in the short to medium term [34,35]. Corticosteroid injections offer certain advantages, as they require less patient effort and involvement; however, they carry risks of complications and adverse effects and have been shown to impair tendon quality and increase the risk of tendon rupture, particularly when repeated [36]. Strengthening exercise programs require greater patient commitment but are virtually risk-free. If confirmed by future studies, the results of the present work suggest that exercise interventions may not only improve clinical symptoms but also optimize tissue quality.

Despite these strengths, several limitations of the present study should be acknowledged. First, this study represents a secondary analysis of an ongoing randomized controlled trial and was not prospectively registered as an independent study; therefore, the findings should be interpreted as exploratory and hypothesis-generating. In addition, the relatively small sample size and the absence of an a priori sample size calculation for SWE outcomes limit the generalizability of the findings. Accordingly, the observed associations did not reach statistical significance, and the study should be considered exploratory and hypothesis-generating, requiring confirmation in larger samples. If a consistent association between increased supraspinatus muscle stiffness and clinical improvement is demonstrated in future research, SWE could potentially be considered an objective and independent marker of a favorable response to exercise therapy, allowing comparisons between different exercise programs and contributing to the optimization of exercise prescription parameters.

Second, all SWE measurements were performed by a single, non-blinded assessor, which may have introduced measurement bias. Although this approach reduced inter-observer variability in this operator-dependent technique, no additional reproducibility analysis was conducted within this study, despite the fact that the reliability of both shear wave elastography and isometric shoulder strength measurements has been well established in previous research. To minimize measurement variability, standardized protocols were applied, and all assessments were performed under consistent technical conditions by a trained and experienced evaluator. Future studies should include blinded assessors and formal reliability analyses to further improve methodological robustness.

Finally, the absence of complementary structural imaging, such as MRI, represents an additional limitation. Structural imaging could have provided further insight into muscle composition, including fatty infiltration or muscle cross-sectional area. Although this was beyond the scope of the present study, future research combining SWE with structural imaging techniques may help to better elucidate the biological meaning of stiffness changes following therapeutic exercise.

## 5. Conclusions

Strength gains following strengthening exercise programs in RCRSP are small and of limited clinical relevance. In contrast, increased supraspinatus muscle stiffness detected by SWE was observed after the exercise intervention and may reflect exercise-related adaptations; however, its biological meaning should be interpreted with caution. In addition to improving shoulder pain and function, strengthening exercise programs in RCRSP may be associated with changes in supraspinatus muscle properties, potentially contributing to clinical improvement.

## Figures and Tables

**Table 1:** Baseline demographic and clinical characteristics of the study participants (N = 39).

Group	Total N = 39		
Variable	n	Mean	SD
Age, years	39	55.6	10.2
BMI	39	27.3	4.4
Sex, Male	14	35.9%	
Education			
Primary	10	25.6%	
Secondary	14	35.9%	
University	15	38.5%	
Pain side			
Left	18	46.2%	
Right	21	53.8%	
Pain side equal dominant side	20	51.3%	
Previous PT	2	5.1%	
Previous analgesic	20	51.3%	
Function			
SPADI	39	52.9	20.5
Pain			
NPRS rest	39	3.5	2.5
NPRS move	39	5.9	2.0
NPRS night	39	6.0	2.8

Abbreviations: BMI, body mass index; N, number of participants; NPRS, Numerical Pain Rating Scale; PT, physical therapy; SD, standard deviation; SPADI, Shoulder Pain and Disability Index.

**Table 2:** Descriptive values of supraspinatus muscle elasticity and strength before and after the exercise program.

N = 39		Pre-Treatment Measures		Post-Treatment Measures	
		Mean	SD	Mean	SD
SWE-R (m/s)	Measure 1	3.75	0.65	3.92	0.79
	Measure 2	3.90	0.90	3.85	0.81
	Measure 3	3.70	0.74	3.96	0.79
	Mean	3.78	0.70	3.91	0.71
Normality (N, %)	<p25	6	16%	5	14%
	normal	14	38%	13	35%
	>p75	17	46%	19	51%
SD (m/s)	Measure 1	0.55	0.23	0.57	0.27
	Measure 2	0.57	0.29	0.61	0.32
	Measure 3	0.56	0.28	0.54	0.30
	Mean	0.56	0.21	0.57	0.23
SWE-C (m/s)	Measure 1	6.89	1.24	7.09	1.03
	Measure 2	7.11	1.04	7.37	0.92
	Measure 3	7.26	1.22	7.56	1.12
	Max	7.82	1.02	8.03	0.94
Normality (N.%)	<p25	9	24%	6	16%
	normal	10	27%	11	30%
	>p75	18	49%	20	54%
SD (m/s)	Measure 1	2.15	0.78	2.11	0.71
	Measure 2	2.07	0.89	1.95	0.73
	Measure 3	2.07	0.74	2.18	0.81
	Max	2.73	0.70	2.62	0.65
Strength (kg)	Measure 1	5.41	3.61	5.79	2.96
	Measure 2	5.79	4.01	6.11	3.26
	Measure 3	5.42	3.56	6.24	3.23
	Mean	5.54	3.62	6.05	3.08

Abbreviations: SWE-C, muscle elasticity during contraction; SWE-R, muscle elasticity at rest; kg, kilograms; Max, maximum value; m/s, meters per second; SD, standard deviation.

**Table 3:** Time effect on supraspinatus shear wave elastography measures and muscle strength.

	Time Effect, *p*-Value	Estimated Marginal Mean (SE)			Difference from Baseline		
		Time	Mean	SE	Diff	CI95%	
SWE-R (m/s)	0.105	Pre	3.78	0.11			
		Post	3.91	0.11	0.12	−0.03	0.28
SWE-C (m/s)	0.285	Pre	7.82	0.16			
		Post	8.03	0.15	0.21	−0.17	0.59
Strength (kg)	0.209	Pre	5.54	0.58			
		Post	6.05	0.49	0.51	−0.28	1.30

Abbreviations: CI, confidence interval; kg, kilograms; m/s, meters per second; SE, standard error; SWE, shear wave elastography; Pre, baseline; Post, post-treatment.

**Table 4:** Time-by-group effects on supraspinatus muscle elasticity and strength stratified by perceived improvement in pain and function.

		Time × Group Interaction (*p*-Value)	Improvement					No Improvement				
			Mean	SE	Difference from Baseline			Mean	SE	Difference from Baseline		
					Mean	CI95%				Mean	CI95%	
**NPRS rest**												
SWE-R (m/s)	Baseline	0.393	3.87	0.14				3.65	0.19			
	Post-treatment		4.03	0.16	0.16	−0.05	0.36	3.68	0.16	0.03	−0.22	0.29
SWE-C (m/s)	Baseline	0.595	7.83	0.23				7.89	0.26			
	Post-treatment		8.10	0.18	0.27	−0.25	0.79	7.94	0.28	0.05	−0.57	0.68
Strength (kg)	Baseline	0.520	5.09	0.60				5.77	1.11			
	Post-treatment		5.89	0.59	0.79	−0.28	1.86	6.01	0.89	0.24	−1.06	1.54
**NPRS move**												
SWE-R (m/s)	Baseline	0.244	3.59	0.13				4.09	0.19			
	Post-treatment		3.76	0.13	0.17	0.04	0.31	4.07	0.21	−0.01	−0.30	0.27
SWE-C (m/s)	Baseline	0.144	7.72	0.20				8.08	0.30			
	Post-treatment		8.13	0.18	0.40	−0.09	0.90	7.88	0.30	−0.20	−0.83	0.44
Strength (kg)	Baseline	0.611	5.21	0.62				5.67	1.19			
	Post-treatment		5.94	0.65	0.74	−0.18	1.65	5.93	0.82	0.26	−1.33	1.85
**NPRS night**												
SWE-R (m/s)	Baseline	0.128	3.73	0.15				3.85	0.17			
	Post-treatment		3.92	0.17	0.19	0.01	0.37	3.82	0.14	−0.02	−0.24	0.19
SWE-C (m/s)	Baseline	0.569	7.89	0.21				7.82	0.29			
	Post-treatment		7.96	0.18	0.07	−0.38	0.53	8.14	0.29	0.32	−0.41	1.06
Strength (kg)	Baseline	0.576	5.09	0.65				5.82	1.10			
	Post-treatment		5.85	0.64	0.76	−0.12	1.65	6.07	0.83	0.25	−1.33	1.82
**SPADI**												
SWE-R (m/s)	Baseline	**0.036**	3.68	0.19				3.86	0.14			
	Post-treatment		3.93	0.22	0.25	0.11	0.40	3.83	0.11	−0.02	−0.24	0.19
SWE-C (m/s)	Baseline	0.520	7.67	0.25				8.02	0.22			
	Post-treatment		7.99	0.20	0.32	−0.32	0.96	8.07	0.24	0.05	−0.45	0.56
Strength (kg)	Baseline	0.308	4.95	0.71				5.76	0.91			
	Post-treatment		5.06	0.57	0.11	−0.74	0.96	6.69	0.77	0.93	−0.40	2.27

Adjusted marginal means (± standard error) at baseline and post-treatment are shown for participants classified as improved or not improved based on predefined clinically relevant thresholds for pain (NPRS at rest, movement, and night) and function (SPADI). Differences from baseline (post-baseline) with 95% confidence intervals (CI) and *p*-values for the time × group interaction derived from linear mixed-effects models are reported. Abbreviations: CI, confidence interval; kg, kilograms; m/s, meters per second; NPRS, Numerical Pain Rating Scale; SE, standard error; SPADI, Shoulder Pain and Disability Index; SWE, shear wave elastography.

**Table 5:** Changes in objective measures according to perceived improvement.

	Time-by-Group Effect	Much Better/Totally Recovered					Much Worst/Something Better				
		Mean	SE	Difference from Baseline			Mean	SE	Difference from Baseline		
				Diff	IC95%				Diff	IC95%	
SWE rest, m/s											
Baseline	0.277	3.67	0.14				4.01	0.19			
Post-treatment		3.83	0.16	0.16	−0.01	0.33	3.99	0.11	−0.02	−0.35	0.31
SWE contract, m/s											
Baseline	0.544	7.76	0.18				8.06	0.36			
Post-treatment		8.03	0.18	0.27	−0.26	0.79	8.05	0.33	−0.01	−0.91	0.88
Strength. kg											
Baseline	0.649	5.56	0.60				5.02	1.33			
Post-treatment		6.27	0.67	0.71	−0.27	1.68	5.26	0.68	0.24	−1.87	2.34

Comparison of elastography measures (SWE at rest, SWE during contraction) and muscle strength between groups with high perceived improvement (“much better” or “totally recovered”) and low or no improvement (“somewhat better” or “much worse”). Values are expressed as means and changes from baseline with 95% confidence intervals (95% CI). Abbreviations: SE, standard error; SWE rest, muscle elasticity at rest; SWE contract, muscle elasticity during contraction; Diff, Difference from baseline; IC95%, 95% confidence intervals; kg, kilograms; m/s, meters per second.

## Data Availability

The raw data supporting the conclusions of this article will be made available by the authors upon reasonable request.

## Supplementary Materials

The following supporting information can be downloaded at: https://www.mdpi.com/article/10.3390/jcm15031015/s1. Table S1: Standardized exercise programs used during the intervention (Programs 1–6); Table S2: Adherence to the exercise program according to clinical and perceived improvement. Figure S1: Representative exercise examples from the most frequently used intervention program (Program 3); Figure S2: Example of the patient exercise diary used to monitor adherence; Figure S3: Heat map of adherence to the exercise program during follow-up; Figure S4: Mean adherence to the exercise program across follow-up weeks; Figure S5: Ultrasound imaging of the supraspinatus tendon in normal conditions and in tendinopathy; Figure S6: Patient positioning and experimental setup during data acquisition; Figure S7: Digital dynamometer used to quantify isometric glenohumeral abduction strength; Figure S8: Shear wave elastography of the supraspinatus muscle at rest (A) and during isometric muscle contraction (B).

## Abbreviations

The following abbreviations are used in this manuscript:

BMI	Body Mass Index
CERT	Consensus on Exercise Reporting Template
CI	Confidence Interval
CONSORT	Consolidated Standards of Reporting Trials
IQR	Interquartile Range
ISCIII	Instituto de Salud Carlos III
kg	Kilograms
Max	Maximum value
MRI	Magnetic Resonance Imaging
m/s	Meters per second
N	Number of participants
NPRS	Numeric Pain Rating Scale
PGI-I	Patient Global Impression of Improvement
PT	Physical Therapy
RCT	Randomized Controlled Trial
RCRSP	Rotator Cuff-Related Shoulder Pain
ROI	Region of Interest
SD	Standard Deviation
SE	Standard Error
SPADI	Shoulder Pain and Disability Index
SWE	Shear Wave Elastography
SWE-C	Shear Wave Elastography during contraction
SWE-R	Shear Wave Elastography at rest
US	Ultrasound

## References

[1] Lucas J., Van Doorn P., Hegedus E., Lewis J., Van Der Windt D. (2022). A systematic review of the global prevalence and incidence of shoulder pain. BMC Musculoskelet. Disord..

[2] Maxwell C., Robinson K., McCreesh K. (2020). Understanding Shoulder Pain: A Qualitative Evidence Synthesis Exploring the Patient Experience. Phys. Ther..

[3] Requejo-Salinas N., Lewis J., Michener L.A., La Touche R., Fernández-Matías R., Tercero-Lucas J., Camargo P.R., Bateman M., Struyf F., Roy J.-S. (2022). International physical therapists consensus on clinical descriptors for diagnosing rotator cuff related shoulder pain: A Delphi study. Braz. J. Phys. Ther..

[4] Lewis J., Mintken P.E., McDevitt A.W. (2025). What’s in a Name? The Case for Using “Rotator Cuff–Related Shoulder Pain” in Clinical Practice. J. Orthop. Sports Phys. Ther..

[5] Desmeules F., Roy J.-S., Lafrance S., Charron M., Dubé M.-O., Dupuis F., Beneciuk J.M., Grimes J., Kim H.M., Lamontagne M. (2025). Rotator Cuff Tendinopathy Diagnosis, Nonsurgical Medical Care, and Rehabilitation: A Clinical Practice Guideline. J. Orthop. Sports Phys. Ther..

[6] Doiron-Cadrin P., Lafrance S., Saulnier M., Cournoyer É., Roy J.-S., Dyer J.-O., Frémont P., Dionne C., MacDermid J.C., Tousignant M. (2020). Shoulder Rotator Cuff Disorders: A Systematic Review of Clinical Practice Guidelines and Semantic Analyses of Recommendations. Arch. Phys. Med. Rehabil..

[7] Powell J.K., Lewis J., Schram B., Hing W. (2024). Is exercise therapy the *right* treatment for rotator cuff-related shoulder pain? Uncertainties, theory, and practice. Musculoskelet. Care.

[8] Powell J.K., Schram B., Lewis J., Hing W. (2022). “You have (rotator cuff related) shoulder pain, and to treat it, I recommend exercise.” A scoping review of the possible mechanisms underpinning exercise therapy. Musculoskelet. Sci. Pract..

[9] Malliaras P., Johnston R., Street G., Littlewood C., Bennell K., Haines T., Buchbinder R. (2020). The Efficacy of Higher Versus Lower Dose Exercise in Rotator Cuff Tendinopathy: A Systematic Review of Randomized Controlled Trials. Arch. Phys. Med. Rehabil..

[10] Clausen M.B., Hölmich P., Rathleff M., Bandholm T., Christensen K.B., Zebis M.K., Thorborg K. (2021). Effectiveness of Adding a Large Dose of Shoulder Strengthening to Current Nonoperative Care for Subacromial Impingement: A Pragmatic, Double-Blind Randomized Controlled Trial (SExSI Trial). Am. J. Sports Med..

[11] Vila-Dieguez O., Heindel M.D., Zipser M.C., Mortazavi K., Kulig K., Bashford G., Mack W., Michener L.A. (2025). Relationship Between Tendon Tissue and Shoulder Disability Change During an 8-Week Exercise Intervention for Rotator Cuff Tendinopathy: An Observational Study. Phys. Ther..

[12] Østerås H., Myhr G., Haugerud L., Torstensen T.A. (2010). Clinical and MRI findings after high dosage medical exercise therapy in patients with long lasting subacromial pain syndrome: A case series on six patients. J. Bodyw. Mov. Ther..

[13] Brage K., Juul-Kristensen B., Hjarbaek J., Boyle E., Kjaer P., Ingwersen K.G. (2020). Strain Elastography and Tendon Response to an Exercise Program in Patients With Supraspinatus Tendinopathy: An Exploratory Study. Orthop. J. Sports Med..

[14] Zhou J., Yang D.B., Wang J., Li H.Z., Wang Y.C. (2020). Role of shear wave elastography in the evaluation of the treatment and prognosis of supraspinatus tendinitis. World J. Clin. Cases.

[15] Hatta T., Giambini H., Uehara K., Okamoto S., Chen S., Sperling J.W., Itoi E., An K.-N. (2015). Quantitative assessment of rotator cuff muscle elasticity: Reliability and feasibility of shear wave elastography. J. Biomech..

[16] Pérez-Porta I., García-Pérez F., Pérez-Manzanero M.Á., Urraca-Gesto M.A., Araujo-Narváez A., Velasco-Arribas M., Navarro-Santana M.J., Plaza-Manzano G., Pérez-Fernández E., Flórez-García M.T. (2025). Adding Multimedia Animations to Exercise Therapy Provides No Additional Benefit for Rotator Cuff–Related Shoulder Pain: A Randomized Clinical Trial. J. Clin. Med..

[17] Pérez-Porta I., Flórez-García M.T., García-Pérez F., Fernández-Matías R., Pérez-Manzanero M.Á., Araujo-Narváez A.M., Urraca-Gesto M.A., Fernández-Lagarejos C., Plaza-Manzano G., Pérez-Fernández E. (2024). Effects of a web application based on multimedia animations to support therapeutic exercise for rotator cuff-related shoulder pain: Protocol for an open-label randomised controlled trial. BMJ Open.

[18] Kim K., Hwang H.J., Kim S.G., Lee J.H., Jeong W.K. (2018). Can Shoulder Muscle Activity Be Evaluated With Ultrasound Shear Wave Elastography?. Clin. Orthop. Relat. Res..

[19] Rosskopf A.B., Ehrmann C., Buck F.M., Gerber C., Flück M., Pfirrmann C.W.A. (2016). Quantitative Shear-Wave US Elastography of the Supraspinatus Muscle: Reliability of the Method and Relation to Tendon Integrity and Muscle Quality. Radiology.

[20] Romero-Franco N., Fernández-Domínguez J.C., Montaño-Munuera J.A., Romero-Franco J., Jiménez-Reyes P. (2019). Validity and reliability of a low-cost dynamometer to assess maximal isometric strength of upper limb: Low cost dynamometry and isometric strength of upper limb. J. Sports Sci..

[21] Pérez-Porta I., Bueno-Horcajadas Á.L., García-Pérez F., Martínez-Ponce D.C., Corrales-Mantecón S., Flórez-García M.T., Velasco-Arribas M. (2025). Normative Data of Supraspinatus Muscle Shear Wave Elastography in Healthy Shoulders: A Cross-Sectional Study. J. Clin. Med..

[22] Wun A., Kollias P., Jeong H., Rizzo R.R., Cashin A.G., Bagg M.K., McAuley J.H., Jones M.D. (2021). Why is exercise prescribed for people with chronic low back pain? A review of the mechanisms of benefit proposed by clinical trialists. Musculoskelet. Sci. Pract..

[23] Runhaar J., Holden M.A., Hattle M., Quicke J., Healey E.L., van der Windt D., Dziedzic K.S., van Middelkoop M., Bierma-Zeinstra S., Foster N.E. (2023). Mechanisms of action of therapeutic exercise for knee and hip OA remain a black box phenomenon: An individual patient data mediation study with the OA Trial Bank. RMD Open.

[24] Powell J., Wood L., Cashin A.G., Lewis J.S. (2025). It is not all about strength: Rethinking mechanistic assumptions in exercise-based rehabilitation for musculoskeletal pain relief. Br. J. Sports Med..

[25] Torstensen T.A., Meen H.D., Stiris M. (1994). The Effect of Medical Exercise Therapy on a Patient With Chronic Supraspinatus Tendinitis. Diagnostic Ultrasound—Tissue Regeneration: A Case Study. J. Orthop. Sports Phys. Ther..

[26] Agergaard A.S., Svensson R.B., Malmgaard-Clausen N.M., Magnusson S.P. (2024). Clinical Outcomes and Tendon Structure at 3- to 4-Year Follow-up After Exercise-Based Treatment of Patellar Tendinopathy: A Prospective Study. Orthop. J. Sports Med..

[27] Deng J., Runhaar J., Breda S.J., Oei E.H.G., Eygendaal D., De Vos R.J. (2025). Do physical or imaging changes explain the effectiveness of progressive tendon loading exercises? A causal mediation analysis of athletes with patellar tendinopathy. J. Sci. Med. Sport.

[28] van Ark M., Rio E., Cook J., van den Akker-Scheek I., Gaida J.E., Zwerver J., Docking S. (2018). Clinical Improvements Are Not Explained by Changes in Tendon Structure on Ultrasound Tissue Characterization After an Exercise Program for Patellar Tendinopathy. Am. J. Phys. Med. Rehabil..

[29] Dubé M.-O., Ingwersen K.G., Roy J.-S., Desmeules F., Lewis J., Juul-Kristensen B., Vobbe J., Jensen S.L., McCreesh K. (2024). Do therapeutic exercises impact supraspinatus tendon thickness? Secondary analyses of the combined dataset from two randomized controlled trials in patients with rotator cuff-related shoulder pain. J. Shoulder Elb. Surg..

[30] Ersever E.M., Goktas B. (2025). The effect of core exercises on shoulder rotator strength, core endurance and suprasipinatus structure in tennis players with rotator cuff injuries. J. Tissue Viability.

[31] Jeong J.Y., Khil E.K., Kim A.Y., Lee S.A., Choi J.A. (2022). Utility of Preoperative Shear-Wave Elastography of the Supraspinatus Muscle for Predicting Successful Rotator Cuff Repair: A Prospective Observational Study With MRI Correlation. Am. J. Roentgenol..

[32] Jeong J.Y., Khil E.K., Seo W. (2024). Prospective Evaluation of Normal Supraspinatus Muscle Using Shear Wave Elastography: Comparison With Symptomatic Tendon Tears. J. Ultrasound Med..

[33] Jiang L., Yu Q., Zhang X., Wang D., Chen H., Jiang W. (2023). Regional assessments of supraspinatus muscle stiffness in normal adults using shear wave elastography. Heliyon.

[34] Lazzarini S.G., Buraschi R., Pollet J., Bettariga F., Pancera S., Pedersini P. (2025). Effectiveness of Additional or Standalone Corticosteroid Injections Compared to Physical Therapist Interventions in Rotator Cuff Tendinopathy: A Systematic Review and Meta-Analysis of Randomized Controlled Trials. Phys. Ther..

[35] Karanasios S., Baglatzis G., Lignos I., Billis E. (2023). Manual Therapy and Exercise Have Similar Outcomes to Corticosteroid Injections in the Management of Patients With Subacromial Pain Syndrome: A Systematic Review and Meta-Analysis. Cureus.

[36] Lin C.-Y., Huang S.-C., Tzou S.-J., Yin C.-H., Chen J.-S., Chen Y.-S., Chang S.-T. (2022). A Positive Correlation between Steroid Injections and Cuff Tendon Tears: A Cohort Study Using a Clinical Database. Int. J. Environ. Res. Public Health.

