# Supplementary material for: Mechanism of Action of Therapeutic Exercise in Rotator Cuff Tendinopathy: What Does Elastography Add?
Source: J Clin Med. 2026 Jan 27;15(3):1015. doi: 10.3390/jcm15031015 (PMC12897793; doi:10.3390/jcm15031015)

SUPPLEMENTARY MATERIALS

Supplementary Material S1: Detailed Exercise Protocol **................................ 1**

Supplementary Material S2: Longitudinal adherence to the exercise program during the 24-week intervention **.. 3**

Supplementary Material S3: Detailed Ultrasound Protocol **........................... 6**

Supplementary Material S4: Detailed SWE Protocol **..................................... 7**

Supplementary Material S5: Isometric Strength Testing Protocol **................. 10**

Supplementary Material S1: Detailed Exercise Protocol

All patients followed a therapeutic exercise program originally developed and validated in a previous clinical trial (BMJ Open 2024)[16,17]. For this elastography substudy, a brief overview of the exercise intervention is provided to contextualize the measurement procedures. The full protocol, including all CERT elements, detailed exercise descriptions, progressions, materials, and adherence procedures, is available in the cited publication.

Summary of the Exercise Intervention

All patients were instructed to perform a progressive exercise program for rotator cuff and scapular stabilizers. The program was delivered by two physiotherapists with >5 years of experience in therapeutic exercise for shoulder pain. Patients attended five in-person sessions on alternate days during the first three weeks, followed by two additional review and progression sessions at weeks 6 and 12.

The program included progressive strengthening of the rotator cuff (abductors and external rotators) and scapular musculature (protractors and retractors), with posterior capsule stretching added when range-of-motion limitations were present. Exercise progression was based on symptom-guided thresholds (≤4/10 pain during execution) or perceived exertion (≥6/10 when pain-free).

All subjects received printed instructions; participants in the experimental arm had additional access to self-explanatory exercise videos through a secured webpage. The intervention was originally reported following the Consensus on Exercise Reporting Template (CERT) to ensure full reproducibility.

Exercise Program Structure: Six standardized combinations of six exercises were available. Physiotherapists selected the most suitable program for each patient. Most participants followed Program 3, and a minority followed Program 5 (which included posterior capsule stretching).

The complete list of exercises included in each program is shown in Supplementary

Table S1. Standardized exercise programs used during the intervention (Programs 1–6).

Exercises included in each of the six standardized intervention programs. An "X" indicates that the exercise was included in the corresponding program.

Exercises	Program					
	1	2	3	4	5	6
Horizontal row		X	X	X	X	X
Supine scapular protraction		X	X	X	X	X
Scaption	X	X	X		X	X
External rotation	X	X	X		X	X
Internal rotation		X				X
Posterior capsule stretching				X	X	X

A complete, fully detailed description of the exercise intervention—including individual exercise fiches, equipment, series, repetitions, progressions, safety criteria, and adherence monitoring—was published previously: BMJ Open 2024[16,17].

Figure S1. Representative exercise examples from the most frequently used intervention program (Program 3)

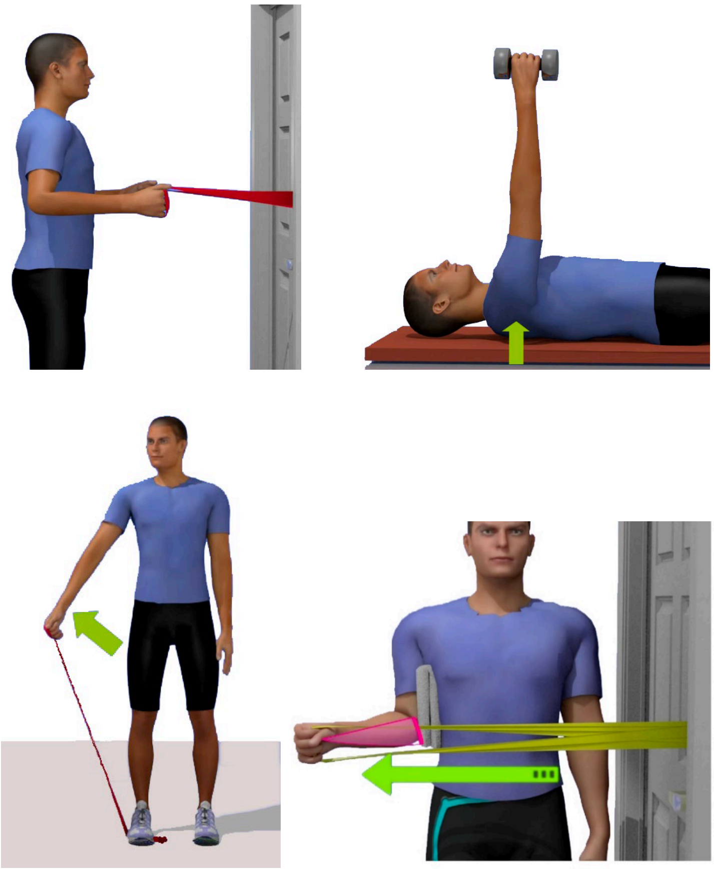

Supplementary Material S2: Longitudinal adherence to the exercise program during the 24-week intervention

Adherence to the exercise program was systematically monitored using a structured patient exercise diary. Participants prospectively recorded each completed exercise session throughout the 24-week intervention period, and diaries were reviewed at each follow-up assessment. This procedure allowed longitudinal tracking of individual exercise exposure and overall adherence patterns over time.

To complement the summary data reported in the main manuscript, this Supplementary Material includes:

(i) a representative example of the exercise diary provided to participants,

(ii) detailed visualizations of adherence over time, including group-level adherence trends, and

(iii) individual adherence patterns displayed as a heat map.

Figure S2. Example of the patient exercise diary used to monitor adherence

Representative page of the structured exercise diary provided to participants (Spanish and English versions). Patients were instructed to mark each day on which the prescribed exercises were performed and to leave blank those days without exercise. Weekly pain intensity was also recorded using a numerical rating scale. Diaries were reviewed at each follow-up visit to prospectively monitor adherence throughout the 24-week intervention period.

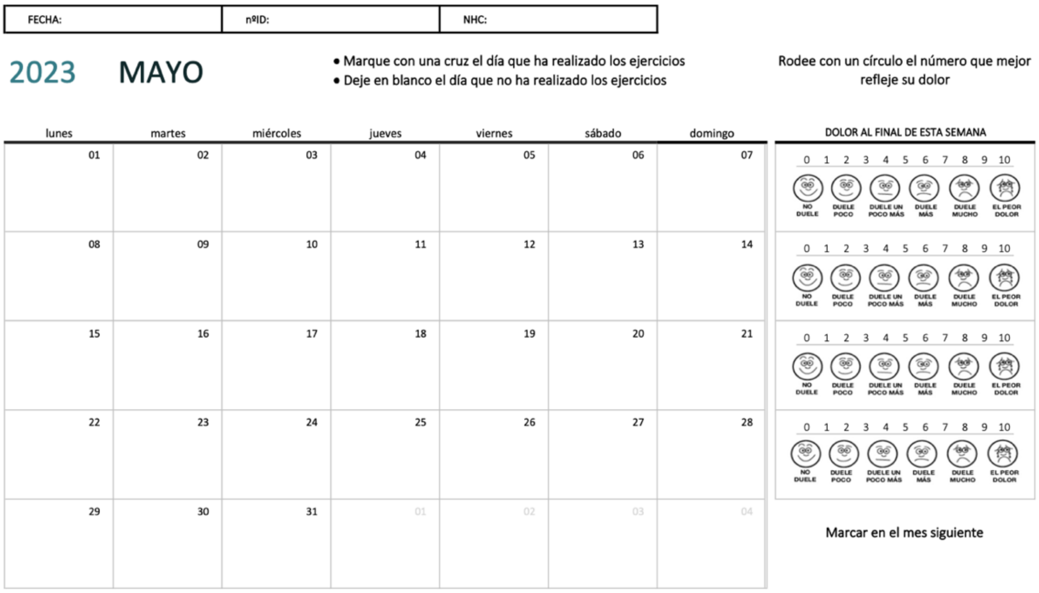
FECHA: | nºID: | NHC:

2023 MAYO

• Marque con una cruz el día que ha realizado los ejercicios
• Deje en blanco el día que no ha realizado los ejercicios

Rodee con un círculo el número que mejor refleje su dolor

lunes	martes	miércoles	jueves	viernes	sábado	domingo	DOLOR AL FINAL DE ESTA SEMANA
01	02	03	04	05	06	07	0 1 2 3 4 5 6 7 8 9 10 NO DUELE / DUELE POCO / DUELE UN POCO MÁS / DUELE MÁS / DUELE MUCHO / EL PEOR DOLOR
08	09	10	11	12	13	14	0 1 2 3 4 5 6 7 8 9 10 NO DUELE / DUELE POCO / DUELE UN POCO MÁS / DUELE MÁS / DUELE MUCHO / EL PEOR DOLOR
15	16	17	18	19	20	21	0 1 2 3 4 5 6 7 8 9 10 NO DUELE / DUELE POCO / DUELE UN POCO MÁS / DUELE MÁS / DUELE MUCHO / EL PEOR DOLOR
22	23	24	25	26	27	28	0 1 2 3 4 5 6 7 8 9 10 NO DUELE / DUELE POCO / DUELE UN POCO MÁS / DUELE MÁS / DUELE MUCHO / EL PEOR DOLOR
29	30	31	01	02	03	04	Marcar en el mes siguiente

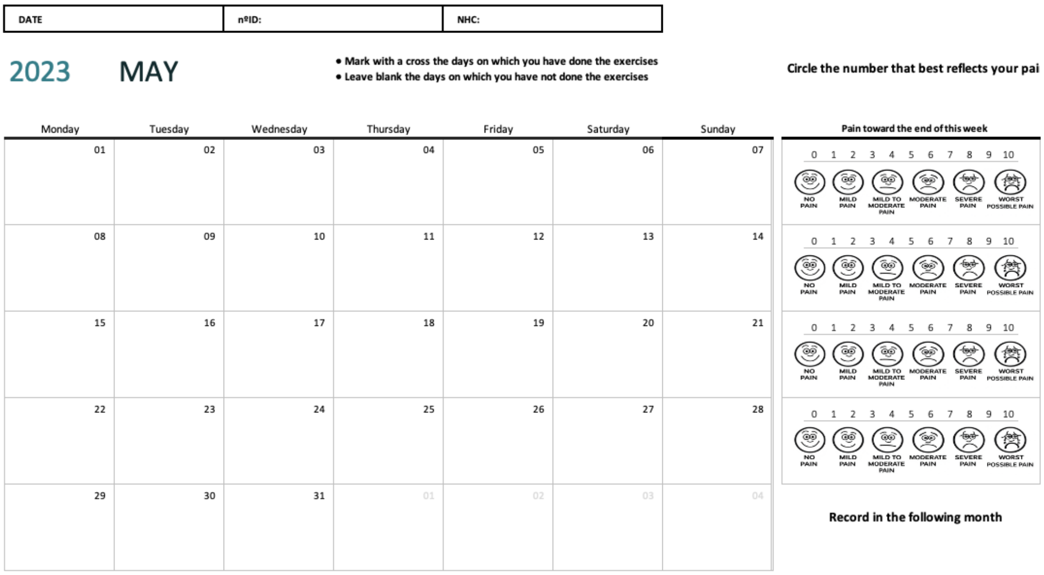

Figure S3. Heat map of adherence to the exercise program during follow-up

Each row represents an individual participant, and each column corresponds to a follow-up week (weeks 1–24). Colors indicate the level of weekly adherence, expressed in arbitrary units (AU), with lighter tones reflecting higher adherence and darker tones indicating lower adherence. Grey cells denote missing data or weeks without recorded adherence. This heat map illustrates inter-individual variability and temporal changes in adherence throughout the intervention period.

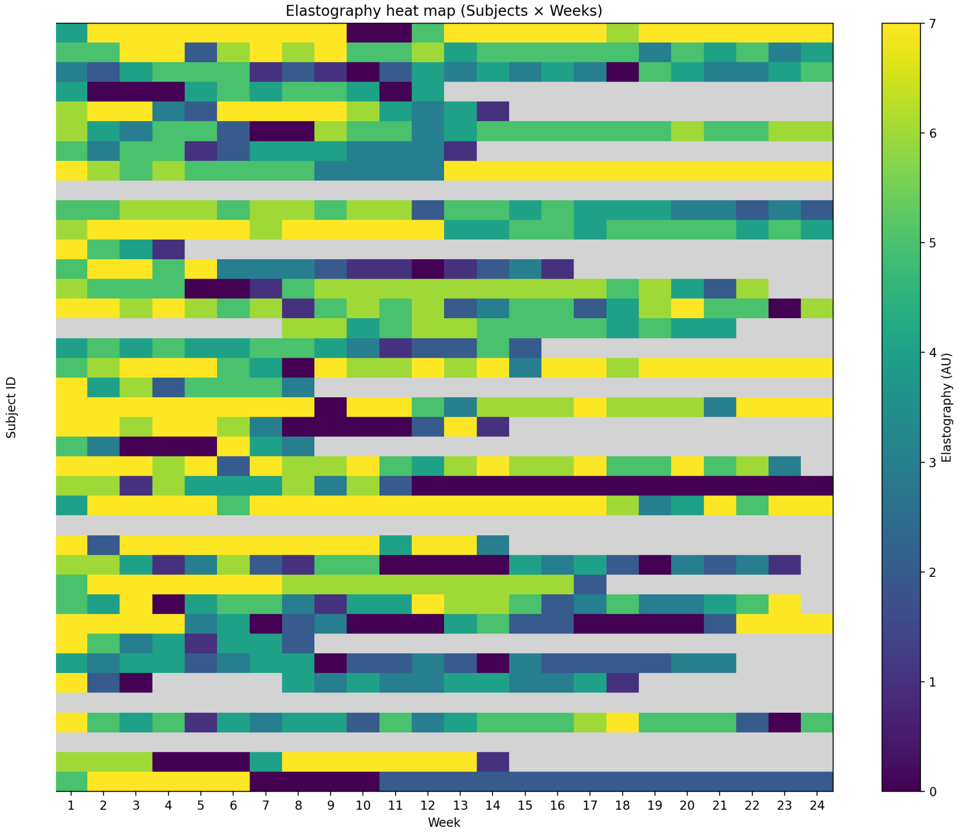

Figure S4. Mean adherence to the exercise program across follow-up weeks

Mean adherence values are shown at the predefined follow-up time points corresponding to weeks 3 (T3), 6 (T6), 12 (T12), and 24 (T24) of the intervention. Adherence was relatively high at early follow-up (week 3), progressively declined up to week 12, and showed a slight recovery at week 24, although values did not return to early follow-up levels.

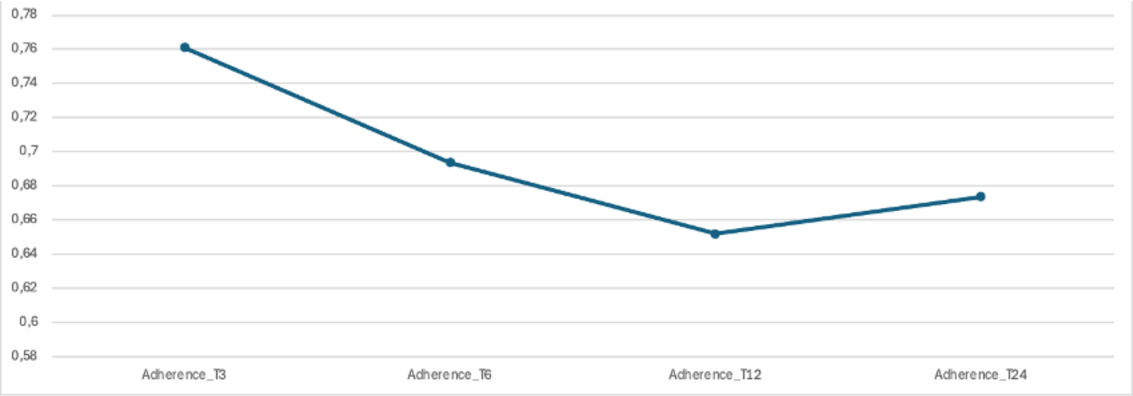

Table S2. Adherence to the exercise program according to clinical and perceived improvement

Adherence at weeks 3, 6, 12, and 24 was 77.6%, 71.0%, 59.4%, and 69.0%, respectively, with a median overall adherence of 68% (interquartile range [IQR]: 54%–83%). No statistically significant differences in adherence were observed between groups according to either clinical improvement or perceived improvement.

	Mediana	Q1	Q3	p-valor
**NPRS rest**				
No improvement	73.7%	51.1%	90.1%	.971
Improvement	68.5%	57.9%	83.1%	
**NPRS move**				
No improvement	67.4%	49.3%	84.7%	.410
Improvement	74.7%	59.8%	85.6%	
**NPRS night**				
No improvement	73.3%	51.3%	90.7%	.942
Improvement	67.9%	60.6%	82.1%	
**SPADI**				
No improvement	69.0%	48.7%	89.1%	.560
Improvement	70.8%	60.6%	83.1%	
**Perceived Improvement**				
Much better/Totally recovered	66.8%	57.9%	81.5%	.647
Much worst-Something better	81.3%	51.1%	89.1%	

Supplementary Material S3: Detailed Ultrasound Protocol

Shoulder ultrasound examinations were performed by the same musculoskeletal radiologist with more than 25 years of experience (A.L.B.H.), using a Canon Aplio i600 ultrasound system (Canon Medical Systems Corporation, Tustin, CA, USA) equipped with a 14-MHz linear transducer. A standardized shoulder musculoskeletal preset was used according to the manufacturer's recommendations.

A systematic evaluation of the main periarticular structures and tendons was performed in both longitudinal and transverse planes.

The supraspinatus tendon was assessed first, with the patient's arm positioned in the Crass position (hand placed behind the lumbar spine), allowing optimal visualization of the tendon footprint. Tendon thickness, echogenicity, fiber continuity, presence of calcifications, and subacromial–subdeltoid bursal fluid were evaluated.

The infraspinatus and teres minor tendons were examined with the patient's hand placed on the contralateral hip and the shoulder internally rotated. Partial- and full-thickness tears, fiber integrity, and degenerative changes were assessed.

The subscapularis tendon was evaluated with the arm in external rotation and the elbow flexed at 90°. The assessment included the tendon insertion on the lesser tuberosity and fiber continuity.

The long head of the biceps tendon was examined within the bicipital groove, with the arm in slight external rotation and the forearm in supination. Fiber continuity and the presence of peritendinous fluid were documented. Dynamic assessment was performed to identify possible subluxation or dislocation of the tendon.

For each structure, at least two static images per plane (longitudinal and transverse) were acquired. All images were archived in a Picture Archiving and Communication System (PACS) (Centricity™ Universal Viewer, GE Healthcare, Chicago, IL, USA).

Figure S5. Ultrasound imaging of the supraspinatus tendon in normal conditions and tendinopathy.

Long- (A) and short-axis (B) ultrasound images of a normal supraspinatus tendon. Long- (C) and short-axis (D) ultrasound images of supraspinatus tendinopathy, showing increased tendon heterogeneity (>) and cortical bone irregularity (arrow). The long head of the biceps tendon is indicated by an asterisk (*).

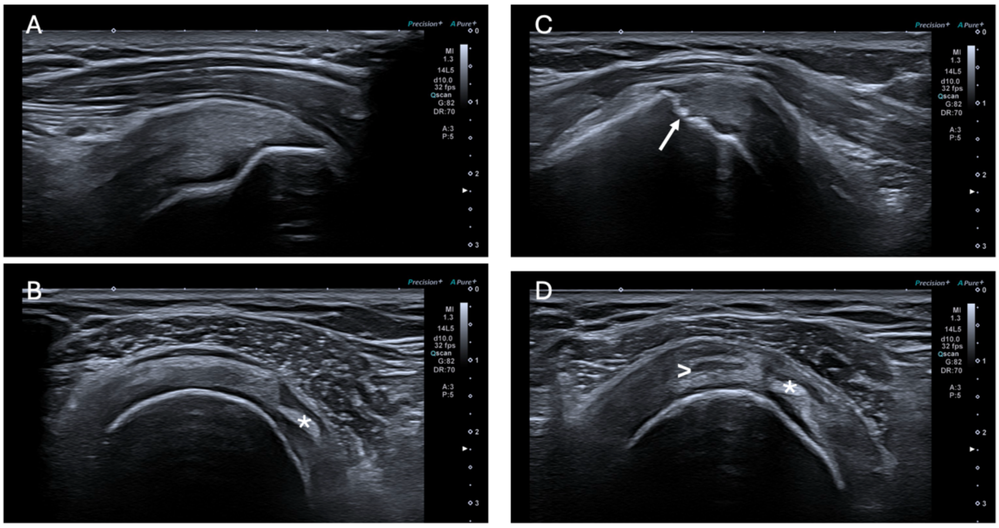

Supplementary Material S4: Detailed SWE Protocol

Technical Principles and Configuration Parameters of Shear Wave Elastography (SWE)

In shear wave elastography, tissue stiffness is estimated from the propagation velocity of the shear wave (Vc), which is related to Young's modulus (E) by the following equation:

$$E = 3 \cdot \rho \cdot Vc^2$$

where ϱ represents tissue density (approximately 980–1100 kg/m^3). A unitary value was assumed for practical purposes.

This relationship is more reliable in isotropic media. Given that skeletal muscle is anisotropic, all measurements were standardized and aligned with the longitudinal axis of the muscle fibers.

Supraspinatus muscle stiffness was assessed using SWE with the same ultrasound system and a 14-MHz linear transducer in musculoskeletal mode. The shear wave velocity range was set to 10 m/s.

A circular region of interest (ROI) of size 5 was used, corresponding to a mean area of approximately 20 mm^2 in this system. This size was considered representative of the muscle belly without exceeding its anatomical boundaries, even in participants with reduced muscle thickness. The ROI was positioned in the central portion of the muscle belly, at its thickest region, aligned with the longitudinal fiber axis, and avoiding visible connective tissue or intramuscular tendon.

The system simultaneously provided a grayscale B-mode anatomical image and two elastography displays:

1. A shear wave propagation map, used to assess wave front homogeneity (typically more homogeneous at rest and more heterogeneous during contraction).
2. A color-coded map of shear wave velocity or stiffness (m/s or kPa), over which the ROI was applied to obtain quantitative measurements.

Standardized Shear Wave Elastography Procedure

SWE was performed following a standardized protocol to ensure intra- and inter-observer reproducibility.

1. Patient Positioning and Initial Preparation

Participants were seated on a chair without back support, with the arm relaxed and supported on the ipsilateral thigh and the elbow flexed at 90°. Both patient and examiner movements were minimized to reduce technical variability.

Prior to SWE assessment, all participants underwent conventional ultrasound examination to evaluate the integrity of the supraspinatus tendon and the remaining rotator cuff tendons, recording the presence of partial- or full-thickness tears.

2. Equipment Configuration

SWE was performed using the shoulder musculoskeletal preset, with a maximum velocity range of 100 m/s and a circular ROI of size 5. Shear wave velocity was expressed in meters per second (m/s).

3. Transducer Placement

The linear transducer was positioned:

- Directly on the skin, perpendicular to the surface, with an inclination angle <20°;
- Using abundant coupling gel, avoiding tissue compression that could artificially increase stiffness values;
- Maintaining a skin-to-target distance <6 cm, as greater depths may compromise wave propagation;
- Aligned parallel to the long axis of the supraspinatus muscle fibers in the longitudinal plane, positioned over the thickest portion of the muscle belly, deep to the trapezius muscle.

Special care was taken to avoid inclusion of fascia or adjacent tissue interfaces within the ROI.

4. Measurements at Rest

With the muscle relaxed:

- A single elastography acquisition was obtained, aiming for homogeneous wave propagation and avoiding areas with interstitial septa.
- Measurement depth did not exceed 3 cm from the skin surface.
- Consecutive measurements were repeated until three valid shear wave velocity values were obtained.

5. Measurements During Isometric Contraction

The same procedure was repeated during isometric contraction:

- Measurements were obtained during a 3–5 second isometric contraction with the arm positioned in approximately 85° of abduction in the scapular plane and neutral rotation.
- To standardize positioning, participants were allowed to support the forearm on the ultrasound device while chair height was adjusted accordingly.

At the moment of elastography acquisition, force output was recorded in kilograms (kg) using a dynamometer. Participants were instructed:

- "Push upward as hard as possible."
- "Hold the position without moving."

Three measurements were obtained, and the mean value was calculated for subsequent analyses.

Figure S6. Patient positioning and experimental setup during data acquisition.
(A) Ultrasound/elastography assessment of the shoulder performed with the patient in a seated position, arm relaxed, and the transducer placed over the rotator cuff region by an experienced examiner.
(B) Measurement of shoulder abduction isometric strength with the patient seated, shoulder abducted and elbow extended, using a hand-held dynamometer secured to an external fixation system. Force was recorded during a sustained isometric contraction according to the study protocol.

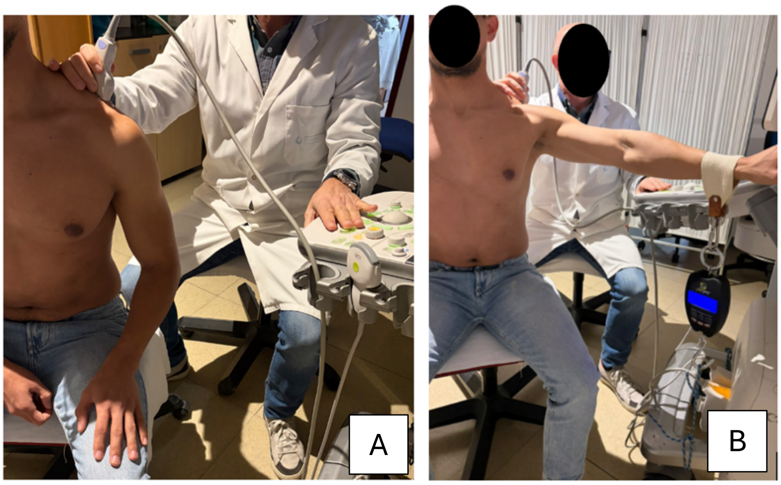

Supplementary Material S5: Isometric Strength Testing Protocol

Isometric Strength Assessment

Isometric glenohumeral abduction muscle strength was quantified using a digital dynamometer (Carp Spirit Water Queen, Bourgogne, France). Strength measurements were performed in the same position used for shear wave elastography (SWE) during muscle contraction, as described in Supplementary Material S3. Force recordings were synchronized with each elastography acquisition.

Participants performed a sustained maximal isometric contraction during the elastography acquisition, and the peak force was recorded in kilograms (kg). No conversion to Newtons was applied. Three measurements were obtained, and the mean value was used for statistical analyses.

Figure S7: Digital dynamometer used to quantify isometric glenohumeral abduction strength

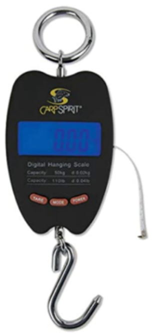

Shear Wave Elastography Measurements

SWE measurements were conducted both at rest and during the previously described isometric contraction. For each acquisition, the ultrasound system provided the mean shear wave velocity (m/s) and the standard deviation within the region of interest (ROI).

Three measurements were obtained under each condition. The final SWE value was calculated using a weighted mean, with the inverse of the standard deviation used as the weighting factor to minimize the influence of measurement dispersion.

Figure S8. Shear wave elastography of the supraspinatus muscle at rest (A) and at isometric muscle contraction (B)

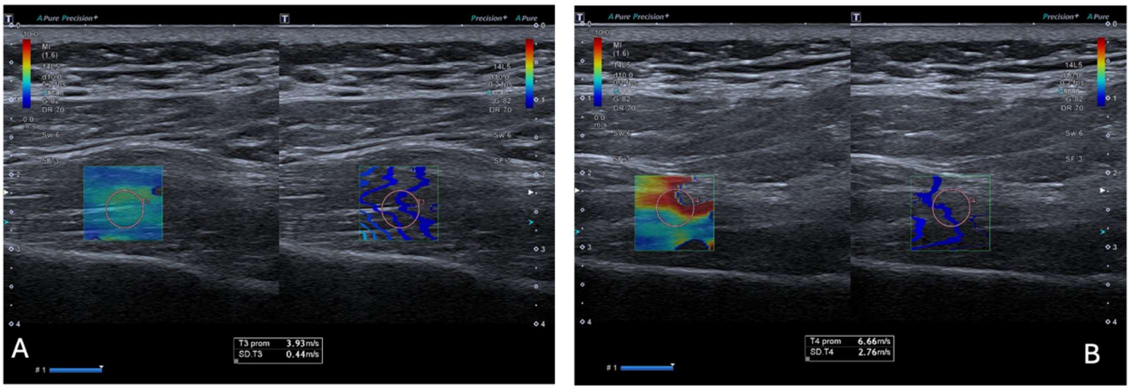

Supplement: Supplementary file 1 [file jcm-15-01015-s001.zip › jcm-4089417-supplementary.pdf]
